# La para-ostéopathie neurogène du coude: résultat de l’arthrolyse chirurgicale (étude rétrospective de 37 cas)

**DOI:** 10.11604/pamj.2019.34.131.16685

**Published:** 2019-11-06

**Authors:** Khaled Khelil, Talel Znagui, Mounira Khezami, Mohamed Achouri, Mounir Hamdi, Lotfi Nouisri

**Affiliations:** 1 Service de Chirurgie Orthopédique et Traumatologique, Hôpital Militaire Principal d’Instruction de Tunis, Tunisie

**Keywords:** Raideur, coude, arthrolyse, para-ostéo-arthropathie-neurogène, calcifications hétérotopiques, Stiffness, elbow, arthrolysis, neurogenic para-osteo-arthropathy, heterotypic calcifications

## Abstract

Les paraostéoarthropathies neurogènes (POAN) sont des ossifications ectopiques, développées à proximité des articulations, qui correspondent à un processus de néo-ostéogénèse ectopique survenant dans les suites de lésions neurologiques centrales ou périphériques, au décours de certains comas (intoxication oxycarbonée, sédation prolongée) et dans les suites de traumatismes périphériques dont les brûlures. Les POAN se localisent quasi-exclusivement au niveau des grosses articulations proximales des membres. L’atteinte du co !ude vient au deuxième rang. Le but de notre étude est d’analyser les résultats de l’arthrolyse chirurgicale de la raideur de 37 coudes séquellaires d’une para ostéo-arthropathie neurogène (POAN) du coude. Il s’agit d’une étude rétrospective qui a porté sur 35 patients et 37 coudes durant une période de 25 ans. Une évaluation préopératoire incluant un examen clinique et un bilan radiologique a été réalisée. Depuis 2003 un arthroscanner du coude était systématiquement demandé. La technique chirurgicale de choix était la résection arthrolyse. Tous les patients avaient bénéficié d’un protocole de rééducation fonctionnelle. Les résultats était analysés à 5 ans de recul moyen (6 mois – 10 ans). Le traumatisme crânien avec coma était la cause de ces POAN dans 58,8%. A l’évaluation préopératoire les raideurs étaient en majorité en flexion (88% des cas), graves ou très graves dans 64,7% des cas. En per-opératoire un secteur fonctionnel allant de -30° d’extension à 130° de flexion était obtenu chez 61,7% des cas et chez 41% à long terme. Le résultat de la libération du nerf ulnaire était satisfaisant dans 92% des cas. Aucune instabilité postopératoire du coude n’a été notée. Une récidive de l’ostéome a été notée chez deux patients présentant des lésions neurologiques définitives. Les résultats étaient équivalents quelque soit le délai de la chirurgie. L’arthrolyse chirurgicale des ostéomes neurogènes du coude est un traitement efficace des ostéomes constitués.

## Abstract (en)

Neurogenic paraosteoarthropathies are ectopic ossifications which develop near the joints. They are a process of neo-ectopic osteogenesis occurring after central or peripheral neurological lesions, in some types of comas (oxygen carbon intoxication, prolonged sedation) and following peripheral traumas including burns. They inolve almost exclusively the large proximal joints of the limbs. Elbow is the second area of involvment. The purpose of our study was to analyze the results of surgical arthrolysis in 37 patients with elbow stiffness due to neurogenic osteoarthropathy of the elbow. We conducted a retrospective study of 35 patients and 37 elbows over a 25-year period. Preoperative assessment included clinical and radiological examination. Since 2003 the patients had undergone systematic elbow arthroscopy. The gold standard surgical treatment was arthrolysis. All patients underwent functional rehabilitation protocol. Outcomes were analyzed after a mean 5-year follow-up period (6 months - 10 years). Neurogenic paraosteoarthropathy was caused by head injury with coma in 58.8% of cases. Preoperative assessment showed bending stiffness in the majority of cases (88%), severe or very severe in 64.7% of cases. Intraoperatively functional elbow range of motion from -30° to 130° was obtained in 61.7% of cases and in 41% of cases in the long term. Ulnar nerve liberation was satisfactory in 92% of cases. No postoperative instability of the elbow was reported. Two patients with definitive neurological lesions had osteoma recurrence. The results were equivalent regardless surgical delay. Surgical arthrolysis is an effective treatment for neurogenic osteomas of the elbow.

## Introduction

Les para-ostéo-arthropathies neurogènes (POAN) correspondent au développement d'os hétérotopique dans les tissus péri articulaires. Elles affectent principalement les grosses articulations mais de fréquence variable en fonction de l'étiologie (hanche en priorité chez les blessés médullaires et épaule ou coude chez les traumatisés crâniens). L’atteinte du coude vient au deuxième rang. Elles ont un retentissement fonctionnel souvent majeur: mobilité du coude et la compression du nerf ulnaire. Le délai opératoire reste un sujet controverse. L’atteinte du nerf ulnaire au niveau du coude est rarement observée dans le cadre de la POAN. Son traitement doit être entrepris de façon précoce, indépendamment de la maturité de l’ostéome. Nous nous proposons d’étudier les résultats de l’arthrolyse chirurgicale de la raideur du coude séquellaire d’une para ostéopathie neurogène.

## Méthodes

Il s’agit d’une étude rétrospective de 37 arthrolyses du coude chez 35 patients présentant une POAN secondaire à un coma prolongé avec séjour en réanimation. L’évaluation préopératoire était clinique et radiologique. Cliniquement l’amplitude articulaire a été évaluée selon les critères de la SOFCOT (Tableau 1). La limitation de la pronation-supination ainsi que la compression du nerf ulnaire (évaluée selon la cotation du British Medical research Council) ont été particulièrement recherchées. Le bilan radiologique comportait des radiographies standards de face et de profil dans tous les cas, une scintigraphie osseuse dans 24 cas et un arthroscanner dans 16 cas. Depuis 2003 l’arthroscanner était systématiquement demandé en préopératoire afin de mieux analyser les rapports de l’ostéome. La classification radio clinique utilisée est celle de Garland (Tableau 2) [1]. L’arthrolyse chirurgicale consistait en une exérèse de l’ostéome avec libération des parties molles et une libération du nerf ulnaire chaque fois qu’il paraît nécessaire. L’évaluation per opératoire a concerné le gain fonctionnel immédiat en amplitudes ainsi que la stabilité finale du coude. L’évaluation post opératoire comportait la mesure de la mobilité articulaire, la cotation du nerf ulnaire ainsi qu’un contrôle radiologique standard afin de dépister une éventuelle récidive.

**Tableau 1:** Valuation de la raideur articulaire selon les critères de la SOFCOT

	STADE I	STADE II	STADE III	STADE IV
Aspect radiologique	Aspect nuageux avec quelques traces d’ossification	Petite mais bien réelle surface d’ossification	Une grande Surface d’ossification	POAN Entièrement ossifiée
Aspect clinique	Aucune limitation articulaire	Aucun retentissement fonctionnel	Diminution des amplitudes articulaires	Ankylose complète

**Tableau 2:** Classification radio clinique de Garland

Raideur	Amplitude articulaire
Très grave	(0 à 30° d’amplitude)
Grave	(31° à 60° d’amplitude)
Modérée	(61° à 90° d’amplitude)
Minime	(amplitude > 90°)

## Résultats

**Figure 1:**
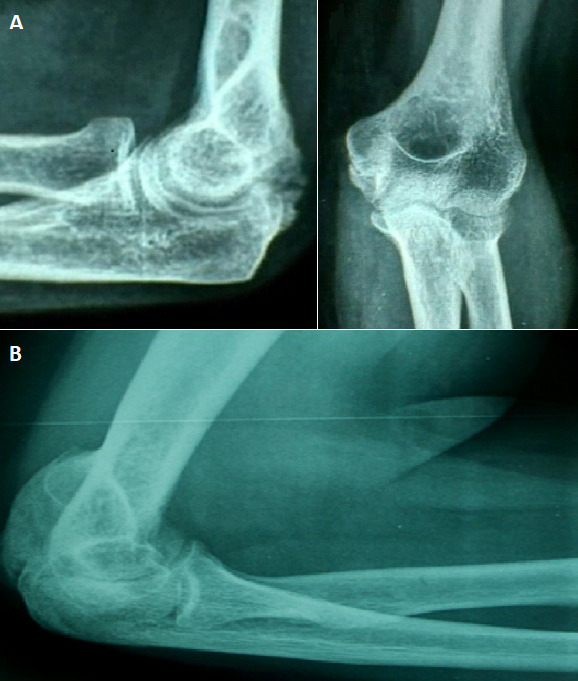
A) ostéome postero-externe; B) ostéome postérieur

Notre série a comporté 37 cas de POAN du coude chez 35 patients d’âge moyen de 29,6 ans avec des extrêmes allant de 21 à 55 ans opérés entre 1987 et 2012 (25 ans) dans le service d’Orthopédie Traumatologie de l’Hôpital Militaire Principal d’Instruction de Tunis. La prédominance masculine était nette (30 hommes et 5 femmes) avec un sex-ratio de 6. Le coude droit était enraidi dans 24 cas contre 13 cas du côté gauche. La raideur a touché une 2^ème^ articulation dans 50% des cas avec par ordre de fréquence la hanche (17 fois dont 7 fois bilatérale), le genou (14 fois dont 4 fois bilatérale) et l’épaule (4 fois dont une fois bilatérale). Chez deux patients, l’atteinte était simultanément bilatérale aux coudes, aux hanches et aux genoux. Sur le plan étiologique, il s’agissait dans tous les cas d’une POAN faisant suite à un coma prolongé secondaire à un traumatisme crânien grave dans 26 cas, une méningite infectieuse dans 7 cas, une intoxication oxycarbonée dans 2 cas et des brulures dans 2 cas. La gravité pour chaque type de raideur appréciée selon les critères établis par la SOFCOT, est résumée dans le Tableau 3. Deux patients avaient une limitation de la pronation-supination. On a noté des paresthésies dans le territoire du nerf ulnaire chez 7 patients avec une cotation moyenne du nerf à S1M3. Les radiographies du coude de face et de profil (Figure 1) ont objectivé un butoir osseux antérieur dans 25 cas, postérieur dans 22 cas et mixte dans 2 cas et des ossifications péri-articulaire dans 21 cas. La plupart des patients (88%) étaient au stade III et IV selon la classification radiologique de Garland.

**Tableau 3:** La gravité pour chaque type de raideur

Raideur	Flexion	Extension	Mixte
Très grave	12	2	-
Grave	7	1	2
Modéree	8	-	1
Minime	3	1	-
Total	30	4	3

**Figure 2:**
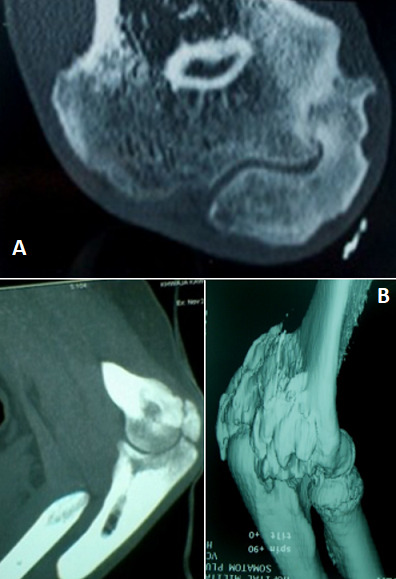
A) coupe tomodensitométrique montrant un pont osseux externe; B) images tomodensitométriques d´un ostéome postérieur

**Figure 3:**
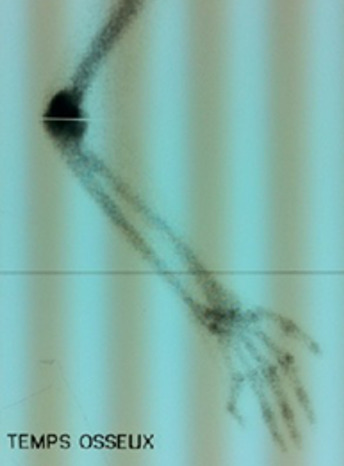
Hyperfixation sur la scintigraphie

**Figure 4:**
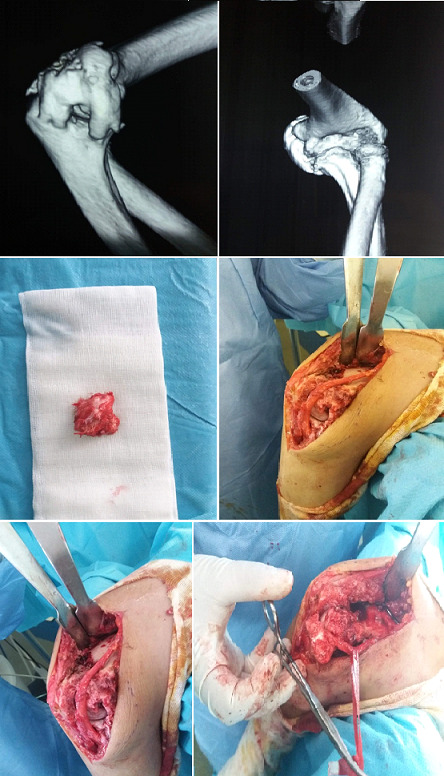
Construction 3D scanner + images per-op: résection ostéome + neurolyse nerf ulnaire

La tomodensitométrie du coude (Figure 2) a mis en évidence des signes typiques (pontage articulaire, englobement du nerf ulnaire). La scintigraphie osseuse (Figure 3) a été faite dans 24 cas (62%), les clichés successifs préopératoires n’ont pas été pratiqués pour évaluer la maturité des ostéomes. Des foyers d’hyperfixations homogènes au niveau des articulations atteintes par l’ostéome ont été mis en évidence. Le délai d’intervention après le début du coma a été en moyenne de 9,2 mois (extrêmes 3 mois et 24 mois). L’arthrolyse (Figure 4) a été menée par voie postéro-latérale 18 fois, interne 6 fois, antéro-latérale 7 fois et antérieur 3 fois. La neurolyse ulnaire simple a été effectuée 12 fois (chez les 7 patients symptomatiques et pour des exigences per opératoire dans 5 cas).la transposition antérieure selon Eaton a été réalisée dans 4 cas. Compte tenu du secteur considéré fonctionnel du coude allant de -30° d’extension à 130° de flexion nous avons évalué le gain fonctionnel immédiat qui correspond aux amplitudes obtenues en per-opératoire, le résultat fonctionnel terminal, apprécié au recul moyen de 5ans (extrêmes de 6 mois à 10 ans) (Tableau 4). La rééducation est débutée le 1^er^ jour sur arthromoteur par une mobilisation passive contrôlée dans le secteur fonctionnel du coude (-30 d’extension et 130° de flexion ou plus) ensuite le patient est adressé au service de rééducation pendant au moins 4 mois. Les suites opératoires ont été simples dans la majorité des cas, les principales complications étaient: l’infection post opératoire dans un cas jugulé par antibiothérapie, des paresthésies résiduelles dans le territoire du nerf ulnaire dans un seul cas. Et la récidive dans 2 cas (au recul moyen de 5 ans) ayant imposé la reprise de l’arthrolyse avec un résultat jugé passable.

**Tableau 4:** Le résultat fonctionnel terminal

	Résultat per-operatoire (%)	Résultat terminal (%)
Flexion >= 130°	2	17
Déficit d’extension <= 130°	(56,8%)	(45,9%)
Flexion < 130°	9	9
Déficit d’extension <= 130	(24,2%)	(24,3%)
Flexion >= 130°	3	6
Déficit d’extension > 30°	(8,1%)	(16,2%)
Flexion < 130°	4	5
Déficit d’extension > 30°	(10,8%)	(13,5%)

## Discussion

Dans la plupart des séries les POAN du coude sont au 2^ème^ rang. Leur topographie est le plus souvent postérolatérale, sus- et rétro-olécranienne, débordant vers le sillon du nerf ulnaire, parfois antérieure limitant alors non seulement la flexion extension mais encore la pronation-supination. La localisation antérieure est beaucoup plus rare, elle ressemble au classique ostéome du brachial, avec un pont osseux complet ou incomplet tendu de la face antérieur de l’humérus au processus coronoïde de l’ulna.

L’analyse radiologique est souvent difficile, le cliché de face est gêné par le blocage en flexion. La lésion, habituellement peu volumineuse est souvent mal visible sur les clichés de profil et de ¾. Le scanner est devenu un examen de routine dans le cadre du bilan préopératoire car il permet de visualiser des POAN faiblement ou non encore calcifiées, de localiser avec précision l’ossification para articulaire au sein des parties molles et permet d’en préciser les bases d’implantation de l’ostéome, son retentissement sur l’articulation (degré de minéralisation, état de l’interligne), ses rapports avec les éléments vasculaires et nerveux (signes indirects de compression du nerf ulnaire par comblement du sillon du nerf ulnaire).

La scintigraphie osseuse a été longtemps utilisée car permettant de poser le diagnostic précoce et pour évaluer la maturité de la POAN et ainsi guider le timing chirurgical. Mais les images scintigraphiques peuvent se négativer en 6 à 12 semaines tout comme la fixation des radiomarqueurs peut se poursuivre au-delà de d’un an et demi. Le stade de maturation scintigraphique ne semble pas corrélé au risque de récidive. De plus certaines lésions continuent à fixer le radioélément plusieurs années après chirurgie de résection même en l’absence de signe clinique de récidive. Cet examen ne peut donc raisonnablement guider le timing opératoire La maturation osseuse (normalité des phosphatases alcalines, de l’hydroxyprolinurie ainsi que refroidissement complet à la scintigraphie) ne semble pas déterminante pour juger du moment de l’opérabilité des patients. Dans différentes séries de la littérature, le pourcentage de récidive n’est modifié ni par les critères de maturation osseuse ni par les critères radiologiques (P. Denormandie *et al.* [2], Rigaux *et al.* [3], Benezech-Lefevre *et al.* [4], Chantraine et Minaire [5]).

Un électromyogramme est demandé s’il existe un doute clinique ou radiographique de compression nerveuse (nerf ulnaire principalement). Une compression nerveuse, même si elle est uniquement électrique, représente une indication chirurgicale précoce de neurolyse du nerf associée à l’ablation de l’ostéome. Cette attitude est partagée par d’autres auteurs (Keenan *et al.* [6], G. Sorriaux *et al.* [7]). La souffrance du nerf et de ses effecteurs moteurs et sensoriels oblige à une prise en charge thérapeutique rapide. C’est pourquoi, notre attitude thérapeutique est chirurgicale et précoce pour libérer et protéger le nerf ulnaire par une neurolyse - transposition antérieure. La transposition antérieure du nerf doit être largement utilisée pour éviter la tension du nerf [8, 9]. Concernant le délai opératoire nous pensons comme d’autres auteurs tels que Roberts [10] et Ippolito [11] qu’aucune différence significative n’existe entre des patients opérés avant et après 1 an et ceci en terme de gain relatif d’amplitude et de récidives. Cette indication opératoire est guidée aussi par la présence ou non d’une souffrance du nerf ulnaire. L’indication du traitement chirurgical des POAN s’est faite dans toutes les séries aux stades III et IV radio clinique de GARLAND.

La technique chirurgicale la plus utilisée dans la plupart des études est une exérèse-arthrolyse par voie postérieure après repérage du nerf ulnaire et éventuellement neurolyse de celui-ci, ou par voie antérieure après repérage du paquet vasculo-nerveux brachial [12]. La neurolyse du nerf débute par sa dissection en amont et en aval de la masse ectopique jusqu’à l’arcade du muscle flexor carpi ulnaris (arcade d’Osborne), le nerf étant protégé sur un lac avant tout geste d’arthrolyse ou résection (Allieu *et al.* 1989 [13]). D’autres (Gallucci *et al.* 2003 [14]) utilisent une pince Kerrison pour libérer le nerf dans son canal, et ensuite réalisent la résection de l’ostéome. La majorité des auteurs (Flin *et al.* 2002 [15]) s’accordent sur la nécessité de la transposition antérieure du nerf, surtout en absence d’arthrolyse, car le nerf pourrait être pris dans un pont osseux néoformé. Cependant, Sorriaux *et al.* (2005) et Chao *et al.* (2002) jugent inutile de transposer le nerf après neurolyse.

Trois méthodes de transposition ont été décrites (Teoh *et al.* 2003 [16]): sous-cutanée, sous-faciale et sous-musculaire. Chaque technique de transposition nerveuse présente des avantages et des inconvénients. En cas d’activité de la POAN, la transposition sous-cutanée réduit le risque d’une nouvelle compression, mais la position superficielle du nerf (sous-cutané) le rend vulnérable aux traumatismes, surtout chez les sujets maigres (Black *et al.* 2000 [17]). La transposition sous-musculaire expose ultérieurement le nerf à un pont osseux néoformé, source de compression nerveuse (Fikry *et al.* 2004). La transposition sous- faciale, est la méthode qui regroupe les avantages des trois techniques conventionnelles, sans pour autant avoir d’inconvénients majeurs (Chuang et Treciak, 1998).

L’essentiel est de permettre une mobilité optimale du coude en préservant sa stabilité [18]. Parfois la récupération de l’extension est insuffisante et nous pensons comme d’autres auteurs [19] que la capsulotomie antérieure est inutile et confions à la rééducation la récupération de l’extension du coude. Le risque principal après la chirurgie est la récidive des ossifications indésirables, dont les conséquences sont parfois plus sérieuses que celles existants avant l’intervention. Ce risque est majeur en cas de persistance de troubles neurologiques définitifs, sur lesquels il n’ya malheureusement aucun moyen d’action et c’était le cas de nos 2 récidives.

La rééducation reste fondamentale d’abord en passif aidé par arthromoteur puis aussi après la sortie du patient et ce de façon prolongée (jusqu’à plus de 6 mois pour certains de nos patients). La mobilisation sous anesthésie générale dans les premières semaines postopératoires dans le but de récupérer les degrés perdus d’améliorer un secteur stagnant a été pour la plupart des auteurs décevantes [20] et ce, en raison de l’inflammation et de la réaction de défense qu’elle a déclenchée. D’ailleurs, elle ne peut donner que des résultats partiels sur les amplitudes et peut être génératrice de neuropathies ulnaires transitoires [21, 22].

## Conclusion

L’arthrolyse du coude séquellaire d’une POAN doit être proposée chaque fois que la raideur affecte un secteur fonctionnel indispensable, sans oublier sa nécessité en cas de localisations multiples afin de permettre la déambulation et la rééducation quand on libère une hanche et/ou un genou et ce d’autant que le patient est jeune. Cependant l’adhésion du patient à un programme de réadaptation long doit être un préalable, indispensable à toute arthrolyse.

### État des connaissances actuelles sur le sujet

- Les para-ostéo-arthropathies neurogènes sont des complications classiques des affections neurologiques centrales, surtout dans les contextes traumatiques;
- Le délai opératoire est conditionné par la maturation des ostéotomes.

### Contribution de notre étude à la connaissance

- Aucune différence significative n’existe entre des patients opérés avant et après la maturation de l’ostéome et ceci en terme de gain relatif d’amplitude et de récidives.

## Conflits des intérêts

Les auteurs ne déclarent aucun conflit d'intérêts.
